# Effects of the Supplementation of Essential Oil Mixtures on Growth Performance, Nutrient Digestibility, Immune Status and Microbial Community in Weaned Piglets

**DOI:** 10.3390/ani13233697

**Published:** 2023-11-29

**Authors:** Yingying Li, Hongrui Cao, Shuya Zhang, Pengfei Guo, Junmei Zhao, Drangon Zhang, Shuai Zhang

**Affiliations:** 1 State Key Laboratory of Animal Nutrition and Feeding, College of Animal Science and Technology, China Agricultural University, Beijing 100193, China; s20223040733@cau.edu.cn (Y.L.); chr8461@cau.edu.cn (H.C.); zhangshuya@cau.edu.cn (S.Z.); 2 Cargill Animal Nutrition, Tianjin 301700, China; pengfei_guo@cargill.com (P.G.); junmei_zhao@cargill.com (J.Z.); drangon_zhang@cargill.com (D.Z.); 3 National Center of Technology Innovation for Pigs (North China Branch), Ministry of Agriculture and Rural Affairs Feed Industry Center, China Agricultural University, Beijing 100193, China

**Keywords:** essential oil, zinc oxide, fecal microbial communities, weaning piglets

## Abstract

**Simple Summary:**

Plant essential oils possess antibacterial, antioxidant, and anti-inflammatory properties, as well as a natural safety profile, thereby making them a plausible alternative to high-dose zinc oxide (ZnO) in the diets of weaned piglets. This study aimed to evaluate the effects of the supplementation of different essential oil mixtures on growth performance, nutrient digestibility, immune status, and microbial community in weaned piglets. The results of this investigation indicate that the dietary supplementation of a combination of low-dose ZnO with essential oil mixtures can enhance the growth performance and intestinal health of weaned piglets, thereby positively regulating microbial density, lowering the incidence of diarrhea, and fostering a favorable condition for intestinal digestive function. Based on this, it is reasonable to suggest that the inclusion of essential oil mixtures and low-dose ZnO in the diets of weaned piglets can serve as a viable substitute for pharmacological dosages of ZnO.

**Abstract:**

Since essential oils—such as cinnamaldehyde, thymol, carvacrol, and eugenol—have antibacterial, antioxidant, and anti-inflammatory properties, this study aimed to examine the supplementation of different essential oil mixtures together with 1600 mg/kg zinc oxide (ZnO) on growth performance, incidence of diarrhea, serum immune indices, fecal volatile fatty acids, and microflora structure in weaned piglets. A total of 240 weaned piglets (Duroc × Landrace × Yorkshire) with an average body weight of 8.85 ± 0.21 kg were randomly allocated to 30 pens (6 pens per diet, 4 males and 4 females per pen). Five different experimental diets were prepared and administered for 28 days: (i) a control diet (C), a corn–soybean basal diet without antibiotics, ZnO, or a supplementation of growth promoters; (ii) a control diet with 400 mg/kg essential oil mixtures 1 (EOM1); (iii) a control diet supplemented with ZnO at 1600 mg/kg (Z); (iv) a diet incorporating the Z diet with the addition of essential oil mixtures 1 at 400 mg/kg (ZOM1); and (v) a diet incorporating the Z diet with the addition of essential oil mixtures 2 at 400 mg/kg (ZOM2). During day (d) 14–28 and d 1–28 of the experiment, the average daily gain (ADG) in piglets in the ZOM1 and ZOM2 groups were higher (*p* < 0.05) compared to the C group. The diarrhea incidence of the Z, ZOM1, and ZOM2 groups were significantly decreased (*p* < 0.05), and the piglets of the ZOM1 group exhibited the lowest diarrhea incidence throughout the trial period. Additionally, the apparent total tract digestibility (ATTD) of neutral detergent fiber (NDF), acid detergent fiber (ADF), ash, organic matter (OM), and ether extract (EE) were higher than those fed the Z diet, and higher levels of NDF, ADF, and crude protein (CP) were observed in groups other than those fed the ZOM1 diet (*p* < 0.01). On d 14, the pigs fed EOM1 and ZOM2 diets showed a somewhat lower (*p* < 0.1) immunoglobulin G (lgG) level in serum than those fed the C diet. Additionally, the IL-8 level in serum in the ZOM1 group tended to be higher than that in the other groups (*p* < 0.1). The piglets fed the ZOM1 diet showed a tendency of lower (*p* = 0.05) acetate concentration in feces on d 14. Principal co-ordinates analysis (PCoA) showed significant differences (*p* < 0.05) in the composition of fecal microbial communities among the groups. Dietary EOM1 significantly increased the number of fecal bacteroides (*p* < 0.05) and tended to increase the number of *Prevotella* (*p* < 0.1). Therefore, EOM1 combined with 1600 mg/kg ZnO tends to reduce diarrhea incidence, tends to improve the fecal microbial community structure and growth performance of weaned piglets, and has the potential to replace pharmacological dosages of ZnO.

## 1. Introduction

Weaning represents one of the most stressful and challenging events in a pig’s life, which can lead to intestinal and immune system dysfunction, thereby impacting the pig’s health status and growth performance [1]. Over recent decades, sub-therapeutic doses of antibiotics have been successfully used to help relieve weaning stress and improve production efficiency in weaned piglets [2]. Unfortunately, the misuse of antibiotics can lead to both enhanced antimicrobial resistance and increased drug residues in animal products, which constitutes a threat to human health [3]. Reports suggest that more than 700,000 human deaths occur annually worldwide due to infections caused by antibiotic-resistant bacteria [2]. Hence, it is highly necessary to explore feasible alternatives to in-feed antibiotics, considering that, in China, agricultural growth promoters have been banned entirely since 1 January 2021 [4].

Dietary supplementation of 2000–3000 mg/kg zinc oxide (ZnO) can improve the performance of piglets and reduce the weaning diarrhea rate by improving intestinal barrier function, reducing the proliferation of pathogenic bacteria such as *Escherichia coli* and *Salmonella* and enhancing the immune system. However, excessive fecal discharge of ZnO from high doses can contaminate the soil and contribute to drug resistance [5,6]. Therefore, potential alternatives such as plant extracts are often used in the diets of weaned piglets. Most plant extracts approved to be used in animal feed contain major components (essential oils) of cinnamaldehyde, thymol, carvacrol, and eugenol.

Cinnamaldehyde is the primary active constituent of cinnamon, containing the aldehyde (-CHO) functional group capable of supplying hydrogen atoms and exhibiting the ability to chelate metals that can activate numerous signaling pathways, which are the fundamental reasons for its wide-ranging medicinal properties. The antibacterial mechanism of cinnamaldehyde involves increasing the permeability of the bacterial cell membrane through its hydrophobic chemical structure, consequently releasing cellular contents extracellularly and ultimately resulting in bacterial cell death [7]. Cinnamaldehyde can inhibit the cell division process of bacteria by binding to the Filamentous temperature-sensitive protein Z. Simultaneously, it inhibits the biofilm formation of various bacteria, including *E. coli* and *Staphylococcus aureus*. Furthermore, cinnamaldehyde possesses anti-quorum sensing activity, which disrupts the bacterial communication system [8]. Cinnamaldehyde decreases cellular oxidative stress by reducing free radical production and indirectly inducing apoptosis. It upregulates the NF-kB signaling pathway, thus reducing nitrous oxide (NO) production, and downregulates the expression of pro-inflammatory markers involved in NO production, resulting in reduced NO levels and ultimately preventing inflammation [7]. Previous studies have shown that a dietary supplementation of 80 mg/kg cinnamaldehyde enhances the growth performance and meat quality of fattening pigs by increasing immune and antioxidant capacities while decreasing glycolysis capacity [9].

Thymol (2-isopropyl-5-methylphenol) is a naturally volatile monoterpene phenol, which serves as the primary active ingredient in oil extracted from *Thymus vulgaris (L.)* and other plants [10]. It possesses antibacterial, antioxidant, and intestinal health-promoting properties. Thymol exerts its antibacterial effects by disrupting bacterial membranes, inhibiting efflux pumps, preventing the formation and destruction of preformed biofilms, impeding bacterial motility, and inhibiting membrane ATPase (adenosine triphosphatase) [11]. Moreover, it reduces the production of NO, elevates glutathione levels to alleviate oxidative stress, and regulates the gut microbiota and immune system, thereby promoting oxidative metabolism in muscles, enhancing nutrient digestibility, and improving intestinal health [10]. Recent research demonstrates that the inclusion of both flaxseed and thymol in diets significantly enhances the oxidative stability of pig blood and meat in comparison to the control group and the flaxseed group [12].

Carvacrol is a monoterpenoid compound that shares non-selective antibacterial properties similar to thymol [11,13]. Studies have demonstrated that the addition of sublethal levels of carvacrol can positively impact pig intestinal health by reducing the motility and invasion of *Salmonella Typhimurium* in pig epithelial cells [14]. This finding underscores the potential of carvacrol in maintaining intestinal health in pigs.

Eugenol is a phenylpropyl phenolic compound, which serves as the main component of *Syzygium aromaticum (L.)*, exhibiting antibacterial, antioxidant, and anti-inflammatory effects. Its antimicrobial mechanisms involve the inhibition of biofilm formation and a decline in the viability of biofilm-forming cells [15]. Moreover, eugenol aids in oxidative stress reduction and mitigating cell damage by providing hydrogen for free radical elimination. Furthermore, it demonstrates anti-inflammatory properties by inhibiting the synthesis and release of inflammatory factors in the NF-κB signaling pathway [16]. Recent research has highlighted eugenol’s ability to inhibit transmissible gastroenteritis virus (TGEV)-induced pyroptosis in intestinal epithelial cells, thus reducing intestinal injury in piglets by impeding the activation of NLRP3 inflammasomes [17].

However, few studies have focused on the combination effects of the key components above as a replacement of ZnO in weaned piglet diets. Therefore, this current study aimed to test the hypothesis that a dietary supplementation of essential oil mixtures and lower-dose ZnO can effectively replace pharmacological dosages of ZnO by reducing diarrhea prevalence while improving the growth performance of weaned piglets. Additionally, this study aimed to explore the underlying mechanisms from the perspectives of nutrient digestibility, immune status, and intestinal microbial community.

## 2. Materials and Methods

All the procedures used in this study were conducted following the Chinese Guidelines for Animal Welfare and approved by the China Agricultural University Institutional Animal Care and Use Committee (Beijing, China). In this study, we used common feed-grade ZnO, with supplementation at a concentration of 1600 mg/kg, which is below the maximum level allowed for weaned pigs in China [18]. The essential oil mixtures 1 (Trade name: Cinergy) was provided by Cargill Animal Nutrition, Tianjin, 301700, China. The essential oil mixtures 2 was purchased from Feedig Feed Technology Co., Ltd. (Liaoning, China).

### 2.1. Animals, Diets, Housing, and Experimental Design

A total of 240 (120 barrows and 120 gilts) five-week-old healthy weaned pigs [Duroc × (Landrace × Yorkshire)] with an average initial body weight (BW) of 8.85 ± 0.21 kg were raised in the National Feed Engineering Technology Research Center, Ministry of Agriculture Feed Industry Center Animal Testing Base (Hebei, China) in July 2022. The piglets were weighted, selected, and distributed in a randomized complete block design according to the body weight and gender. The five experimental groups assigned were as follows: (i) C (control diet), a corn–soybean basal diet without antibiotics, ZnO, or growth promoter supplementations; (ii) EOM1, a control diet with 400 mg/kg essential oil mixtures 1 (Trade name: Cinergy, combination of 10.0% cinnamaldehyde and 5.0% thymol); (iii) Z, a control diet supplemented with ZnO at 1600 mg/kg; (iv) ZOM1, a diet incorporating the Z diet with the addition of essential oil mixtures 1 at 400 mg/kg; and (v) ZOM2, a diet incorporating the Z diet with the addition of essential oil mixtures 2 (combination of 8.0% cinnamaldehyde, 2.5% thymol, 5.0% carvacrol, and 1.5% eugenol) at 400 mg/kg. Each treatment group had 6 replicated pens, with each pen consisting of 4 barrows and 4 gilts. The basal diet was formulated to satisfy the nutrient requirements, except for Zn, as suggested by the NRC (2012) [19]. The ingredient compositions of the basal diet are listed in Table 1. The proportion of feed ingredients in the treatment groups was consistent with that in the control group, except for corn, which subtracted the proportion of oil mixtures or ZnO in each group. Chromium oxide (3 g/kg) was added to all diets as an indigestible marker to calculate dietary nutrient digestibility.

The study employed an all-in–all-out feeding system in a pig farm, where weaned piglets were accommodated in pens measuring 1.2 × 2.1 m^2^. Each pen was equipped with plastic-slatted floors, an automatic stainless steel nipple drinker, and a three-hole feeder. The initial temperature of the room was kept at 28 °C during the first week and then gradually lowered by 1 °C weekly while maintaining relative humidity at 60% to 70%. To promote a hygienic environment and prevent the occurrence of specific pig diseases, the pens and barn were cleaned twice a week using brooms. Throughout the 28-day study period, all of the piglets were provisioned with ad libitum feed and water.

### 2.2. Sample Collection

The body weight of the individual piglets and the feed consumption by pen were measured on day (d) 0, 14, and 28 to calculate the average daily gain (ADG), average daily feed intake (ADFI), and gain to feed ratio (G:F). Clinical signs of diarrhea were assessed at 09:00 and 16:00 hours each day by observers blinded to treatments, and visual observations were carried out for each piglet by touching the perianal skin and checking the feces on the floor of each pen to obtain better assessments. The diarrhea incidence value was calculated according to the following formula reported by Long et al. [20]:

Diarrhea incidence (%) = the total number of diarrhea pigs/(number of pigs × total observational days) × 100(1)

For each treatment, representative feed samples of 1 kg were collected from the barn. From d 25–27, approximately 200 g of fresh fecal samples were collected from each pen and immediately stored at −20 °C. After this period, the 3-day collection of fecal samples was pooled by pen and then dried for 72 h in a 65 °C drying oven. Both feed and fecal samples were finely ground by a grinder (Beijing Ever Bright Medical Treatment Instrument Co., Ltd., China), until they could pass through a 1 mm screen (40 mesh), before chemical analysis. On Day 14 and 28, using the rectal palpation technique, fresh fecal samples were collected into sterile plastic bags from each pen and immediately stored at −20 °C for the detection of short-chain fatty acids (SCFAs) and calprotectin. Additionally, a total of thirty fresh fecal samples (each sample from one pig close to the average group body weight per pen) of five groups were representatively collected via rectal palpation on Day 28 and immediately immersed in liquid nitrogen and then stored at −80 °C for subsequent microbial composition analysis.

On the morning of Day 14 and Day 28, blood collection was performed after selecting 30 pigs (each sample from one pig per pen) close to the average group body weight, except for the pens with the largest and smallest initial weight. The anterior vena cava puncture technique was used to collect 8 mL of blood for the blood samples, which were then stored in vacuum container tubes (Becton Dickinson Vacutainer Systems, Franklin Lakes, NJ, USA). The blood samples were allowed to clot at room temperature for 30 min and centrifuged (Heraeus Biofu 22 R, Hanau, Germany) at 3000× *g* for 15 min at 4 °C. The serum was then extracted and stored at −20 °C for further analysis.

### 2.3. Analytical Methods

Feed and fecal samples were analyzed for crude protein (CP) (method 984.13), dry matter (DM) (method 930.15), and ash (method 942.05) using the Official Methods of Analysis of AOAC International (2007) [21]. Organic matter (OM) was calculated as the difference between dry matter (DM) and ash. Gross energy (GE) was determined with an Automatic Isoperibol Oxygen Bomb Calorimeter (Parr 6400 Calorimeter, Moline, IL, USA). Neutral detergent fiber (NDF) and acid detergent fiber (ADF) were determined using the fiber bags and fiber analyzer equipment (Ankom Technology, Macedon, NY, USA), according to the procedure of Van Soest et al. (1991) [22]. The NDF content was analyzed using heat-stable amylase and sodium sulfite without correction for insoluble ash. The ADF fraction was expressed with the inclusion of ash. Concentrations of chromium (Cr) were determined via flame atomic absorption spectrophotometry (Z-5000; Hitachi, Tokyo, Japan) after the samples were wet-digested using nitric-perchloric acid (3:1). The formula below was used to calculate the apparent total tract digestibility (ATTD) of the nutrients:

ATTD = 1 − (Nf × Cd)/(Nd × Cf) (2)

where Nf = nutrient concentration in the feces (g/kg), Nd = nutrient concentration in the diet (g/kg), Cd = Cr concentration in the diet (g/kg), and Cf = Cr concentration in the feces (g/kg).

Concentrations of serum immunoglobulins (IgA, IgG, and IgM); cytokines including interleukin-1α (IL-1α), interleukin-1β (IL-1β), interleukin-2 (IL-2), interleukin-4 (IL-4), interleukin-6 (IL-6), interleukin-8 (IL-8), and interleukin-10 (IL-10); tumor necrosis factor-α (TNF-α); interferon-γ (INF-γ); and fecal calprotectin were examined using commercially available ELISA kits and a standard specification microplate reader provided by Beijing Laibo Tailui Technology Development Co., Ltd.(Beijing, China).

To determine the concentration of SCFAs in feces, a modified method from a previous study [23] was used. In detail, around 0.5 g of the fecal sample was dissolved in 8 mL of ultrapure water. After subjecting it to an ultrasound for homogenization, the fecal sample solution was centrifuged at 5000× *g* for 15 min, and the supernatant was then diluted (1:50) and filtered through a 0.20 mm nylon membrane before its injection into a Gas Chromatograph System (Agilent HP 6890 Series, Santa Clara, CA).

Total bacterial genomic DNA was extracted from the fecal samples following the manufacturer’s protocol, using the QIAamp Fast DNA Stool Mini Kit (Qiagen, Hilden, Germany).

To analyze the quality and concentration of DNA, 1.0% agarose gel electrophoresis and a NanoDrop^®^ ND-2000 spectrophotometer (Thermo Fisher Scientific Inc., Waltham, MA, USA) were used. The extracted DNA was kept at −80 °C until further use. Primer pairs 338F (5’-ACTCCTACGGGAGGCAGCAG-3’) and 806R (5’-GGACTACHVGGGTWTCTAAT-3’) were employed to amplify the hypervariable region V3-V4 of the bacterial 16S rRNA gene, using an ABI GeneAmp^®^ 9700 PCR thermocycler (ABI, Carlsbad, CA, USA).

All of the samples were amplified in triplicate, and the PCR product underwent extraction and purification using the AxyPrep DNA Gel Extraction Kit (Axygen Biosciences, Union City, CA, USA) following the manufacturer’s instructions and quantified using Quantus™ Fluorometer (Promega, Santa Clara, CA, USA).

Finally, equimolar amounts of purified amplicons were paired-end sequenced on an Illumina MiSeq PE300 platform by Illumina, San Diego, CA, USA, according to standard protocols provided by Majorbio Bio-Pharm Technology Co., Ltd. (Shanghai, China).

### 2.4. Statistical Analysis

Data on growth performance, diarrhea incidence, nutrient digestibility, serum cytokine markers, fecal calprotectin concentration, and SCFAs concentrations were checked for normality and outliers using the UNIVARIATE procedure of SAS 9.4 (SAS Institute Inc., Cary, NC, USA). The statistical model included dietary treatment as the only fixed effect and block (BW) as the random effect. The data were then analyzed by ANOVA using the GLM procedure of SAS 9.4, and Tukey’s test was used to adjust the multiple comparisons. For the growth performance and diarrhea incidence data, the pen was considered the experimental unit, while the individual pig was regarded as the experimental unit for the other indexes. The values were presented as least square means with standard error of the mean.

For the microbiota sequencing data, an in-house Perl script was used to de-multiplex the raw Illumina fastq files. The quality of the resulting sequences was assessed using the FASTP version 0.19.6 and merged by FLASH version 1.2.7 with overlapping sequences longer than 10 bp and without any mismatches [24]. Next, the optimized sequences were clustered into operational taxonomic units (OTUs) using UPARSE 7.1 [25,26] with a 97% sequence similarity level. The OTU table was manually filtered by removing chloroplast sequences from all samples. To minimize the impact of sequencing depth on alpha and beta diversity measures, the number of 16S rRNA gene sequences from each sample was rarefied to 20,000, which individually yielded an average good coverage of 99.09%. The Ribosomal Database Project (RDP) Classifier version 2.2 was used to analyze the phylogenetic affiliation of each 16S rRNA gene sequence [27].

A bioinformatic analysis of the gut microbiota was conducted using the Majorbio Cloud platform (https://cloud.majorbio.com (accessed on 23 November 2023)). Mothur v1.30.1 was implemented to calculate rarefaction curves and alpha diversity indices (observed OTUs, Ace, Chao, and Shannon indexes) based on the OTUs. β-diversity was visualized using principal coordinate analysis (PCoA) by Vegan v2.5-3 package, with Bray–Curtis dissimilarity distances between the samples. Linear discriminant analysis effect size (LEfSe) was performed to identify significant differences in the relative abundance of phylum to genera taxa among the different groups (Linear discriminant analysis (LDA) score > 3). Gut microbiota composition, based on relative abundance, was analyzed using the Kruskal–Wallis method.

Statistical significance was considered present when *p* < 0.05, while 0.05 ≤ *p* < 0.10 were considered to indicate a trend.

## 3. Results

### 3.1. Growth Performance and Diarrhea Incidence

Growth performance and diarrhea incidence results are presented in Table 2. There were no differences (*p* > 0.05) in ADG among the treatments during Day 1–14. During Day 14–28 and Day 1–28, pigs fed the ZOM1 diet and the ZOM2 diet had higher ADG (*p* < 0.05) than those fed the C diet. Moreover, the pigs in the Z group had higher ADG (*p* < 0.05) than those in the C group during Day 1–28. No significant differences (*p* > 0.05) in ADFI or G:F were observed among the five treatments during the entire trial. The incidence of diarrhea was significantly lower (*p* < 0.01) in the Z, ZOM1, and ZOM2 groups than in the C group throughout the entire trial period.

### 3.2. Apparent Nutrient Digestibility

The apparent nutrient digestibility data is presented in Table 3. On d 28, the ATTD of NDF, OM, GE, and EE in the Z group were significantly lower compared to that in the C group (*p* < 0.01). Moreover, pigs fed the ZOM1 diet had lower ATTD of NDF, OM, GE, and CP (*p* < 0.01) compared to those fed the C diet. Furthermore, no significant differences (*p* > 0.05) were observed in the ATTD of most nutrients in the C, EOM1, and ZOM2 groups, except for the ATTD of ADF, which was significantly lower in the ZOM2 group than that in the C group (*p* < 0.01).

### 3.3. Fecal Calprotectin and Serum Cytokine

The concentrations of fecal calprotectin and serum cytokines are presented in Table 4. On d 14, the serum IgG level in the Z group was slightly lower (*p* = 0.07) compared to the C group. Moreover, the serum IL-8 level in the ZOM1 group was higher than in the other groups, albeit with borderline statistical significance (*p* = 0.06). However, no significant differences were observed in fecal calprotectin concentration and serum hormone levels among all treatment groups on d 28 (*p* > 0.05).

### 3.4. Fecal Short-Shain Acids Concentrations

Table 5 shows that piglets in the ZOM1 group had a tendency to exhibit a lower acetate concentration in feces on d 14 (*p* = 0.05). However, on d 28, no significant differences were observed in the concentration of each kind of short-chain fatty acid in the feces among the five treatment groups (*p* > 0.05).

### 3.5. Fecal Microbial Diversity

**Figure 1:**
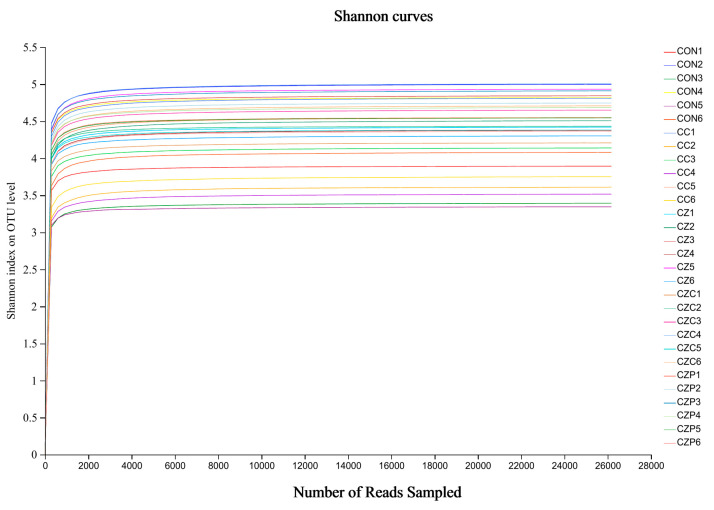
Rarefaction curve for multi-sample operational taxonomic unit (OTU) numbers.

**Figure 2:**
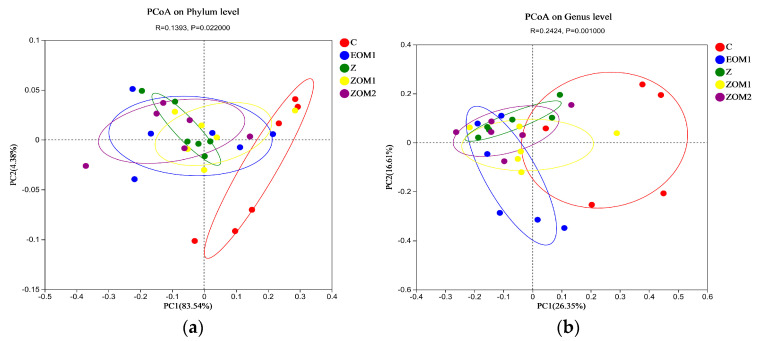
Microbial β-diversity in the feces of weaned piglets on d 28 of the experiment. (**a**) PCoA analysis at phylum level; (**b**) PCoA analysis at genus level. (i) C, control diet; (ii) EOM1, C + 400 mg/kg EOM1 supplement; (iii) Z, C + 1600 mg/kg ZnO; (iv) ZOM1, Z + 400 mg/kg EOM1 supplement; and (v) ZOM2, Z + 400 mg/kg EOM2.

Figure 1 displays the Shannon Diversity Index, which reflects community diversity. The flat curve indicates that the sequencing data quantity was sufficient to reflect most of the microbial diversity information in the samples, indicating that the amount of sequencing data in the samples for analysis was reasonable. PCoA analysis (*p* = 0.022, Figure 2a; *p* = 0.001, Figure 2b) revealed significant differences in the composition of fecal microbial communities among the five treatment groups.

**Figure 3:**
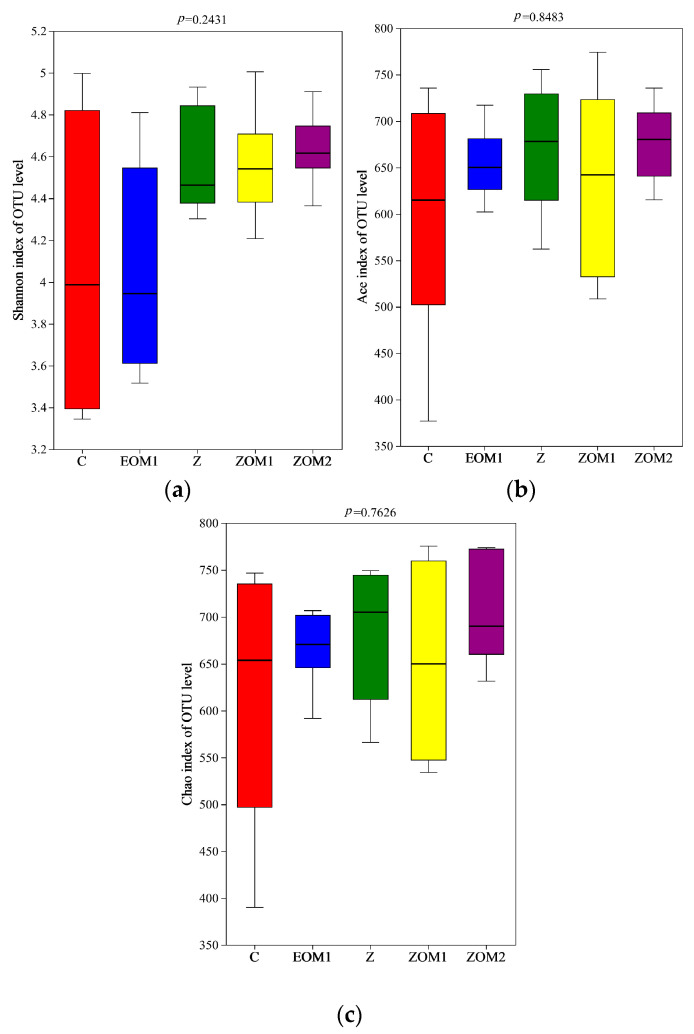
Microbial α-diversity in the feces of weaned pigs on d 28 of the experiment. (**a**) Shannon index; (**b**) Ace index; (**c**) Chao index. (i) C, control diet; (ii) EOM1, C + 400 mg/kg EOM1 supplement; (iii) Z, C + 1600 mg/kg ZnO; (iv) ZOM1, Z + 400 mg/kg EOM1 supplement; and (v) ZOM2, Z + 400 mg/kg EOM2.

The Kruskal–Wallis H test of the OTU diversity index showed that the α-diversity indexes of Shannon, Ace, and Chao did not significantly change (*p* > 0.05, Figure 3). The results of α-diversity and β-diversity together implied that EOM1, Z, ZOM1, and ZOM2 supplementation had a considerable impact on the community composition but not on the overall number of microbial community species in weaned piglets.

**Figure 4:**
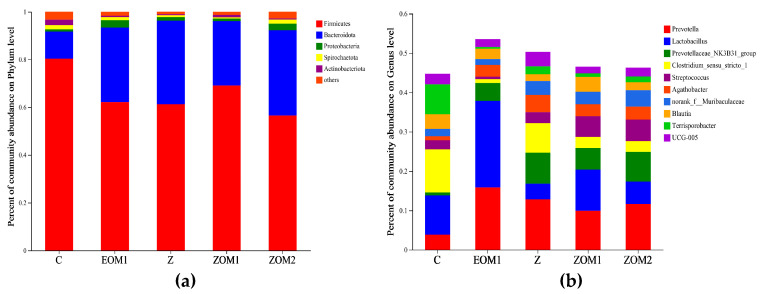
Relative abundance of fecal microbiota at phylum (**a**) and genus (**b**) levels. (i) C, control diet; (ii) EOM1, C + 400 mg/kg EOM1 supplement; (iii) Z, C + 1600 mg/kg ZnO; (iv) ZOM1, Z + 400 mg/kg EOM1 supplement; and (v) ZOM2, Z + 400 mg/kg EOM2.

Figure 4 and Table 6 display the top five fecal microflora at the phylum level and the top ten fecal microflora at the genus level. Z and ZOM2 groups had a significantly lower relative abundance of Firmicutes and Actinobacteriota than the C group (*p* < 0.05), but the relative abundances of Bacteroidota in the EOM1, Z, ZOM1, and ZOM2 groups were significantly higher than in the C group (*p* < 0.05). A statistical analysis of the top ten relative abundance genera (excluding the *unnamed norank_f__Muribaculaceae* and *UCG-005*) showed that the relative abundance of *Prevotella* significantly increased in EOM1, Z, and ZOM2 groups compared to the C group (*p* < 0.05), and the relative abundance of the *Prevotellaceae_NK3B31_group* significantly increased in Z, ZOM1, and ZOM2 groups compared to the C group (*p* < 0.05). However, the relative abundance of *Streptococcus* in the EOM1 group significantly decreased (*p* < 0.05). Therefore, the results indicated that the supplementation of EOM1, Z, ZOM1, and ZOM2 significantly changed the average relative abundance of fecal microorganisms at both the phylum and genus level.

**Figure 5:**
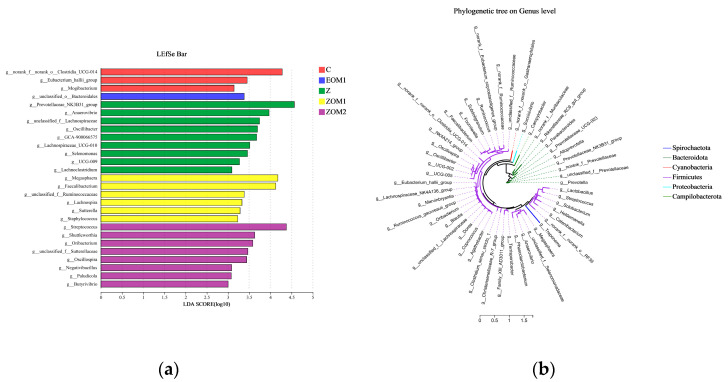
LEfSe discriminant analysis of multilevel species differences and microbial community evolution analysis. (**a**) Indicator bacteria with LDA scores of 3 or greater in fecal bacterial communities (**b**) Cladogram showing the phylogenetic distribution of the fecal bacteria from phylum to genus of weaned piglets. (i) C, control diet; (ii) EOM1, C + 400 mg/kg EOM1 supplement; (iii) Z, C + 1600 mg/kg ZnO; (iv) ZOM1, Z + 400 mg/kg EOM1 supplement; and (v) ZOM2, Z + 400 mg/kg EOM2.

To search for biomarkers with statistical differences among treatment groups, LDA value and LEfSe analysis were performed to search tagged taxon default values greater than 3. As illustrated in Figure 5a, 27 distinct microbial genera were detected among all five treatment groups. The phylogenetic analysis of major groups of related microbial communities was conducted using phylogenetic evolutionary trees. Figure 5b showed that the unclassified bacteria at the genus level with a high abundance in weaned piglets were mainly derived from Firmicutes and Bacteroidota. Moreover, as shown in the figure, *Prevotellaceae_NK3B31_group* and *Prevotella* were categorized under Bacteroidota while *Streptococcus* was classified under Firmicutes.

## 4. Discussion

### 4.1. Effects of Essential Oil Mixture Supplementation on the Performance and Diarrhea Rate of Weaned Piglets

Plant essential oils and high levels (usually 3000 ppm) of ZnO have been reported to improve growth performance, reduce diarrhea rates, and enhance intestinal health in weaned piglets [28,29,30]. However, excessive ZnO supplementation in animal feed is considered a great contamination threat to the environment, and related controlling actions have been taken in many countries and regions [31]. For instance, the excessive use of ZnO in pig production may lead to the leaching of metals (zinc) to fields fertilized with pig slurry and even may pose a risk to aquatic species [32]. And the ZnO or basic zinc chloride level has been set to no more than 1600 mg/kg (calculated as zinc element) in weaned piglet diets in China since 2018. Moreover, the European Union has forbidden the utilization of pharmacological dosages of ZnO in animal feed since June 2022. Therefore, this study mainly investigated the impact of essential oil mixtures as potential replacements for pharmacological dosages of ZnO on growth performance and diarrhea incidence in weaned piglets. This study showed that the pigs fed with the essential oil mixtures and lower ZnO level diets had had higher ADG and lower diarrhea rates than those fed the control diet during d 14–28 and d 1–28, indicating that the simultaneous addition of essential oil mixtures and low-dose ZnO might have a superior effect on the improvement of ADG and diarrhea resistance in weaned piglets. On the other hand, no significant differences were observed in growth performance and diarrhea incidence among the pharmacological dosages of the ZnO group and the combination of different essential oil mixtures and low-dose ZnO groups throughout the experimental period, indicating little synergistic effect between the essential oils used in this study and ZnO on production performance. Similarly, a synergistic effect might not occur between essential oil and antibiotics, as previous findings suggested that the combination of plant preparations (carvacrol and thymol) with antibiotics inhibited the growth-promoting effects of weaned piglets [33]. However, subtle synergies cannot be entirely ruled out, and further evidence is necessary to understand their impact on production performance. Additionally, the recommended dose of essential oil used in this experiment is 400 mg/kg, as advised by the product. Nevertheless, alternative doses should be explored for comparative testing to determine if they have synergistic effects.

### 4.2. Effects of Essential Oil Mixture Supplementation on Nutrient Digestibility in Weaned Piglets

Previous studies have suggested that dietary supplementation of essential oils can improve nutrient digestibility in weaned piglets [34,35] by possibly increasing the secretion and activity of endogenous enzymes and improving intestinal morphology [36]. In this experiment, compared to the high-dose ZnO group, cinnamaldehyde and thymol supplementation significantly enhanced the digestibility of NDF, ADF, ash, OM, GE, and EE. Additionally, the combination of different essential oils (cinnamaldehyde + thymol, or cinnamaldehyde + thymol + carvacrol + eugenol) and low-dose ZnO showed numerically greater NDF, ash, OM, GE, and EE digestibility in piglets compared to the high-dose ZnO group, further proving the positive effect of essential oils on nutrient digestibility. A previous study revealed that an increase in feed intake led to the linearly declined ATTD of nutrients including CP, DM, EE, carbohydrate, and GE in diets with a specific nutrient density [37]. Therefore, the increased feed intake of the ZnO group in this study might explain the correspondingly significantly decreased ATTD values.

### 4.3. Effects of Essential Oil Mixture Supplementation on Serum Immune Indexes and Inflammatory Factors of Weaned Piglets

Immunoglobulins are proteins with antibody activity in the body, which can reflect the active immune capacity of the body [38]. Increasing the concentration of immunoglobulin in pigs can improve their immune function and promote the development of their immune system, so as to relieve weaning stress [39]. This study found that cinnamaldehyde and thymol supplementation in both early and late experimental periods had no significant effects on the serum immune indexes of weaned piglets compared to the control group. However, when compared to the high-dose ZnO supplementation group, the combination of cinnamaldehyde, thymol, and low-dose ZnO supplementation showed a tendency to increase serum IgG and the IL-8 levels of weaned piglets, which was consistent with previous research results [34,40]. IgG is an immunoglobulin abundant in animals as the initiator of immune response, and it has functions such as the agglutination, regulation, and precipitation of antigens [41]. Dietary supplementation of 200 mg/kg plant essential oil (containing 4.5% cinnamaldehyde and 13.5% thymol) has been reported to enhance the immune function of weaned piglets by increasing the levels of serum albumin, IgA, and IgG [42]. IL-8 is a pro-inflammatory chemokine primarily produced by various immune cells and epithelial cells in peripheral tissues, with the primary functions of inducing chemotaxis, facilitating the migration of various immune cells to the infection site, and contributing to angiogenic effects [43,44]. However, the concentration of IL-8 in serum or plasma is often studied as an outcome variable of inflammation [45]. Therefore, the results in this current study suggested that the combination of cinnamaldehyde, thymol, and low-dose ZnO has the potential to enhance the immune function of weaned piglets. Fecal calprotectin is another biomarker that reveals the inflammatory status of the body; however, no significant changes were observed among the five treatment groups, which might be due to the relatively healthy status of the piglets used in this study. Moreover, the piglets chosen for this experiment were older (35 days), limiting the applicability of the experiment’s findings in practical use, since the first two weeks following weaning are typically when medicinal zinc oxide (ZnO) is administered.

### 4.4. Effects of Essential Oil Mixture Supplementation on Short-Chain Fatty Acids in the Feces of Weaned Piglets

Short-chain fatty acids (SCFAs) are the major products from the gut microbial fermentative activity, mainly including acetate, propionate, and butyrate, which can directly activate G-coupled-receptors, inhibit histone deacetylases, and serve as energy substrates [46,47,48]. In addition, short-chain fatty acids can be produced by the fermentation of dietary fiber through various biochemical pathways [46]. During the early stage of this trial, adding combination of cinnamaldehyde, thymol, and low-dose ZnO tended to reduce the acetate content in the feces of weaned piglets compared to the control group, but no significant difference was observed in comparison to high-dose ZnO group, and combination of essential oil mixtures and low-dose ZnO groups. However, no significant difference or trend was observed in the later stage of this animal trial across all groups regarding the content of short-chain fatty acids. In previous studies, dietary supplementation with 400 mg/kg essential oil mixtures (carvacrol, thymol and cinnamaldehyde) had no significant effects on volatile fatty acid concentrations in colon of piglets, and analysis of microbial α-diversity and β-diversity showed little difference [49]. In this study, although short-chain fatty acids concentrations were not affected, microbial diversity was changed. In general, the supplementation of ZnO and essential oils mixtures might not have any significant impact on the regulation of intestinal microbial metabolism in weaned piglets.

### 4.5. Effects of Essential Oil Mixture Supplementation on the Intestinal Microflora of Weaned Piglets

In this study, through the multi-group difference test, it was found that there was no significant difference in the α-diversity index (Shannon, ACE, Chao indexes) among the treatment groups. However, the PCoA map showed that the microflora of piglets with different diets were separately clustered. These findings suggested that the supplementation of ZnO and essential oils mixtures had significant effects on the microbial community composition but not on the overall number of microbial communities in weaned piglets. Among the main bacteria in the intestinal tract of weaned piglets, Bacteroides and Firmicutes had the highest abundance, which was consistent with the results of previous research [50,51].

Weaning easily causes changes in the intestinal flora structure of piglets, resulting in an imbalanced microecology [52]. In this study, the addition of cinnamaldehyde and thymol were found to significantly increase the relative abundance of Bacteroidota compared to the control group, and there were no significant differences compared to the high-dose ZnO group or low-dose ZnO and essential oil mixtures groups. Bacteroidota plays an important role in helping the host decompose polysaccharides to improve nutrient utilization rates [53], accelerating the angioformation of intestinal mucosa [54], developing the immune system, improving the host immunity, and maintaining intestinal microecological balance [55,56]. Recently, it was reported that the low abundance of Bacteroides and its associated metabolic dysfunction might be a microbial marker of physiological postweaning diarrhea in piglets [57]. Moreover, Bacteroides in the intestinal tract usually produce butyrate, which activates T-cell-mediated responses through interactions with the immune system and limits the colonization of potentially pathogenic bacteria in the gastrointestinal tract [58]. Recent studies also revealed that an improved inflammatory response caused a common structural alteration in the intestinal flora, which involved a reduction in the relative abundance of Firmicutes and an elevation in the relative abundance of Bacteroides, some of which were accompanied by decreased Actinobacteria and increased *Prevotella*, which was similar to the results of this study [59,60].

At the generic level, the addition of cinnamaldehyde and thymol significantly increased the relative abundance of *Prevotella* compared to the control group, with no significant difference observed compared to the high-dose ZnO group or low-dose ZnO and essential oil mixtures groups. *Prevotella* reportedly increased in healthy weaned piglets after weaning, as was observed after the comparison of fecal microflora compositions in diarrhea-prone, healthy, and sick weaned piglets [61], which was consistent with our findings. *Prevotella* contributes to the decomposition of proteins and carbohydrates, participates in the degradation of host polysaccharides and amino acid metabolism, and is believed to resist glucose intolerance and promote liver glycogen storage [62]. Previous studies showed that *Prevotella* could indirectly stimulate the production of cytokines, such as IL-1, IL-6, IL-8, and IL-23, through epithelial cells to promote an immune response [63]. In an in vitro study, an individual supplementation of 1000 mg/L cinnamaldehyde, thymol, carvacrol, or eugenol significantly reduced the abundance of *Streptococcus* and *Lactobacillus*, except for eugenol [13]. However, in this study, there were no significant differences in *Lactobacillus* abundance among the treatment groups, while the addition of cinnamaldehyde and thymol alone significantly reduced the relative abundance of *Streptococcus*. These changes in microflora suggested that cinnamaldehyde and thymol supplementation improved the beneficial microflora structure of the distal intestine of piglets, which was equivalent to dietary supplementation with high-dose ZnO and the combination of essential oil mixtures and low-dose ZnO. Nevertheless, this experiment solely focused on the low level of ZnO supplementation; thus, further comparisons with a group receiving pharmacological levels of ZnO should be conducted to assess its potential as a substitute for pharmacological ZnO supplementation.

## 5. Conclusions

In conclusion, the addition of dietary supplements ZOM1 or ZOM2 reduced the incidence of diarrhea, which may be attributed to improvements in the microbial structure. Furthermore, the inclusion of ZOM1 or ZOM2 in the diet could enhance the growth performance of weaned pigs. These findings indicate that the utilization of ZOM1 or ZOM2 as supplements could potentially serve as a viable alternative to pharmacological dosages of ZnO.

## Figures and Tables

**Table 1:** Ingredients and chemical compositions of the basal diet (as-fed basis).

Items	Content
Ingredients, %	Data
Corn	60.66
Soybean meal	14.00
Extruded soybean	5.00
Soy protein concentrate	4.00
Fish meal	3.00
Whey power	5.00
Soybean oil	2.50
Glucose	2.00
Dicalcium phosphate	1.20
Limestone	0.80
Salt	0.15
L-Lysine HCl, 78.8%	0.56
DL-Methionine, 98.5%	0.10
L-Threonine, 98.5%	0.20
L-Tryptophan, 98.5%	0.03
Chromic oxide	0.30
Vitamin-mineral premix ^1^	0.50
Calculated compositions, %	
Digestible energy, kcal/kg	3503
Crude protein	18.59
Calcium	0.80
Available phosphorus	0.41
Standardized ileal digestible amino acid, %	
Lysine	1.36
Methionine	0.42
Threonine	0.81
Tryptophan	0.24

^1^ Vitamin and mineral premix provided the following per kilogram of diet: 12,000 IU vitamin A as vitamin A acetate, 2500 IU vitamin D as vitamin D3, 30 IU vitamin E as dl-α-tocopheryl acetate, 12 μg vitamin B12, 3 mg vitamin K as menadione sodium bisulfate, 15 mg d-pantothenic acid as calcium pantothenate, 40 mg nicotinic acid, 400 mg choline as choline chloride, 30 mg Mn as manganese oxide, 90 mg Fe as iron sulfate, 80 mg Zn as zinc oxide, 10 mg Cu as copper sulfate, 0.35 mg I as ethylenediamine dihydroiodide, and 0.3 mg Se as sodium selenite.

**Table 2:** Effects of ZnO and essential oil mixture supplementation on growth performance and diarrhea incidence in weaned piglets.

Items	Groups					SEM	*p*-Value
	C	EOM1	Z	ZOM1	ZOM2		
Day 1 to 14							
ADG (g/d)	256	293	297	307	296	15.7	0.22
ADFI (g)	496	517	557	547	546	29.3	0.57
G/F	0.51	0.57	0.54	0.56	0.54	0.02	0.32
Diarrhea rate, %	12.77 ^a^	10.71 ^a^	7.14 ^b^	5.43 ^b^	6.03 ^b^	/	<0.0001
Day 15 to 28							
ADG (g/d)	434 ^b^	422 ^b^	504 ^ab^	519 ^a^	531 ^a^	28.4	0.033
ADFI (g)	823	780	908	936	958	53.8	0.13
G/F	0.53	0.54	0.56	0.57	0.56	0.02	0.69
Diarrhea rate, %	7.30 ^a^	5.43 ^ab^	3.04 ^c^	3.49 ^bc^	4.06 ^bc^	/	0.0024
Day 1 to 28							
ADG (g/d)	345 ^c^	357 ^bc^	401 ^ab^	413 ^a^	413 ^a^	18.9	0.042
ADFI (g)	660	649	732	742	752	40.3	0.24
G/F	0.53	0.55	0.55	0.56	0.55	0.01	0.47
Diarrhea rate, %	10.06 ^a^	8.07 ^a^	5.09 ^b^	4.47 ^b^	5.06 ^b^	/	<0.0001

^a–c^ Different superscripts within a row indicate a significant difference (*p* < 0.05). Values are least square means of six replicate pens with four barrows and four gilts per pen per treatment. (i) C, control diet; (ii) EOM1, C + 400 mg/kg EOM1 supplement; (iii) Z, C + 1600 mg/kg ZnO; (iv) ZOM1, Z + 400 mg/kg EOM1 supplement; and (v) ZOM2, Z + 400 mg/kg EOM2; SEM, standard error of the mean; ADG, average daily gain; ADFI, average daily feed intake; G/F, gain to feed ratio.

**Table 3:** Effects of ZnO and essential oil mixture supplementation on apparent total tract digestibility of nutrients in weaned piglets.

Items, %	Groups					SEM	*p*-Value
	C	EOM1	Z	ZOM1	ZOM2		
Day 28 of the experiment							
Neutral detergent fiber	64.01 ^a^	66.21 ^a^	47.57 ^c^	51.82 ^bc^	57.66 ^ab^	0.011	<0.01
Acid detergent fiber	45.73 ^ab^	53.30 ^a^	44.45 ^b^	43.73 ^b^	42.73 ^b^	0.015	<0.01
Ash	36.28 ^ab^	44.90 ^a^	32.27 ^b^	35.01 ^ab^	44.28 ^a^	0.025	<0.01
Organic matter	87.28 ^a^	86.48 ^ab^	83.27 ^c^	83.42 ^bc^	86.04 ^abc^	0.0075	<0.01
Gross energy	84.05 ^a^	83.21 ^abc^	79.49 ^c^	80.06 ^bc^	83.38 ^ab^	0.0090	<0.01
Crude protein	76.16 ^a^	73.44 ^a^	69.50 ^ab^	64.90 ^b^	70.36 ^ab^	0.018	<0.01
Ether extract	68.83 ^ab^	68.27 ^ab^	60.95 ^c^	61.79 ^bc^	70.66 ^a^	0.025	0.03

^a–c^ Different superscripts within a row indicate a significant difference (*p* < 0.05). Values are least square means of six replicate pens with four barrows and four gilts per pen per treatment. (i) C, control diet; (ii) EOM1, C + 400 mg/kg EOM1 supplement; (iii) Z, C + 1600 mg/kg ZnO; (iv) ZOM1, Z + 400 mg/kg EOM1 supplement; and (v) ZOM2, Z + 400 mg/kg EOM2; SEM, standard error of the mean.

**Table 4:** Effects of ZnO and essential oil mixture supplementation on fecal calprotectin concentration and serum cytokines levels in weaned piglets.

Items	Groups					SEM	*p*-Value
	C	EOM1	Z	ZOM1	ZOM2		
Day 14 of the experiment							
CaLP (ug/mg)	39.55	30.86	32.69	38.44	36.58	3.61	0.39
IgA (ug/ml)	10.04	10.23	10.34	10.26	10.54	0.48	0.96
IgG (mg/mL)	7.19	6.57	6.37	7.13	6.52	0.24	0.07
IgM (ug/ml)	9.75	8.96	9.16	9.47	9.08	0.25	0.21
TNF-α (ng/L)	39.73	37.34	38.09	38.37	38.17	1.11	0.63
IL-1α (pg/ml)	0.93	0.96	0.97	0.94	0.96	0.31	0.87
IL-1β (ng/L)	72.85	71.98	71.29	71.77	68.77	3.20	0.92
IL-2 (pg/mL)	31.93	30.69	31.11	31.89	30.47	1.17	0.86
IL-4 (pg/mL)	6.28	6.21	6.22	6.42	5.81	0.39	0.84
IL-6 (ng/L)	34.23	34.69	30.66	33.40	32.84	1.52	0.37
IL-8 (ng/L)	57.94	56.93	59.48	65.19	58.21	2.05	0.06
IL-10 (ng/L)	17.13	16.59	17.10	17.13	16.62	0.46	0.82
IFN-γ (pg/mL)	118.48	114.35	112.38	115.52	113.88	8.29	0.99
Day 28 of the experiment							
CaLP (ug/mg)	28.95	31.15	31.14	32.26	32.64	2.52	0.85
IgA (ug/ml)	12.19	13.45	13.05	12.03	13.07	0.63	0.45
IgG (mg/mL)	6.72	7.04	6.95	6.82	7.33	0.28	0.59
IgM (ug/ml)	9.59	10.02	9.56	9.71	10.12	0.29	0.56
TNF-α (ng/L)	43.05	46.00	44.30	42.89	45.55	1.74	0.62
IL-1α (pg/ml)	1.02	1.10	1.07	1.07	1.13	0.04	0.42
IL-1β(ng/L)	75.79	77.28	73.10	71.62	78.15	3.40	0.63
IL-2 (pg/mL)	33.66	35.58	34.19	33.55	35.14	1.09	0.69
IL-4 (pg/mL)	6.31	6.43	6.29	6.14	6.53	0.28	0.84
IL-6 (ng/L)	36.26	37.97	39.37	37.07	36.22	2.04	0.79
IL-8 (ng/L)	57.87	58.35	61.42	60.35	62.57	2.84	0.74
IL-10 (ng/L)	17.60	17.36	17.56	16.89	18.32	0.51	0.42
IFN-γ (pg/mL)	136.82	156.18	149.41	150.16	157.82	6.96	0.26

Values are least square means of six replicate pens with four barrows and four gilts per pen per treatment. (i) C, control diet; (ii) EOM1, C + 400 mg/kg EOM1 supplement; (iii) Z, C + 1600 mg/kg ZnO; (iv) ZOM1, Z + 400 mg/kg EOM1 supplement; and (v) ZOM2, Z + 400 mg/kg EOM2; SEM, standard error of the mean; CaLP, calprotectin; IgA, immunoglobulin A; IgG, immunoglobulin G; IgM, immunoglobulin M; TNF-α, tumor necrosis factor-α; IL-1α, interleukin-1α; IL-1β, interleukin-1β; IL-2, interleukin-2; IL-4, interleukin-4; IL-6, interleukin-6; IL-8, interleukin-8; IL-10, interleukin-10; IFN-γ, Interferon-γ.

**Table 5:** Effects of ZnO and essential oil mixture supplementation on short-chain fatty acids concentrations in the feces of weaned piglets.

Items, ug/g	Groups					SEM	*p*-Value
	C	EOM1	Z	ZOM1	ZOM2		
d 14 of the experiment							
Acetate	7675.43	6920.10	7138.91	6182.07	6497.23	350.89	0.053
Propionate	4395.54	3794.34	3610.47	3540.80	4166.76	298.49	0.23
Isobutyrate	427.48	424.26	435.09	367.35	482.70	59.81	0.76
Butyrate	2928.61	2460.52	2580.76	1855.40	2815.72	331.84	0.21
Isovalerate	747.31	747.97	751.00	660.04	833.78	117.67	0.89
Valerate	960.34	877.98	964.71	807.33	887.28	149.29	0.94
d 28 of the experiment							
Acetate	5497.98	5755.44	5924.16	5866.20	5383.86	313.53	0.68
Propionate	2910.83	3071.81	3070.91	2833.64	3021.30	199.47	0.90
Isobutyrate	265.99	342.12	338.38	287.86	264.14	54.09	0.74
Butyrate	1794.79	1652.72	2116.39	1781.70	1907.13	199.80	0.37
Isovalerate	409.02	526.77	539.96	456.36	417.32	96.08	0.81
Valerate	459.43	605.61	628.08	446.73	620.32	68.95	0.96

Values are least square means of six replicate pens with four barrows and four gilts per pen per treatment. (i) C, control diet; (ii) EOM1, C + 400 mg/kg EOM1 supplement; (iii) Z, C + 1600 mg/kg ZnO; (iv) ZOM1, Z + 400 mg/kg EOM1 supplement; and (v) ZOM2, Z + 400 mg/kg EOM2; SEM, standard error of the mean.

**Table 6:** Comparison of differences in the main phyla and genera of fecal flora in each group.

Items, %	Groups					SEM	*p*-Value
	C	EOM1	Z	ZOM1	ZOM2		
Phylum							
Firmicutes	80.33	62.16	61.25	69.10	56.52	6.12	0.085
Bacteroidota	11.38 ^b^	31.27 ^a^	35.02 ^a^	26.95 ^a^	35.72 ^a^	4.84	0.014
Proteobacteria	0.91	3.05	1.48	1.07	2.78	0.85	0.55
Spirochaetota	1.80	1.22	0.79	0.53	1.68	0.73	0.83
Actinobacteriota	2.24 ^a^	0.63 ^b^	0.47 ^b^	1.00 ^ab^	0.48 ^b^	0.33	0.047
Firmicutes/Bacteroidota	7.06	1.99	1.75	2.56	1.58		
Genus							
*Prevotella*	3.79	15.88	12.83	9.94	11.64	2.74	0.066
*Lactobacillus*	10.05	21.96	4.01	10.46	5.73	3.87	0.45
*Prevotellaceae_NK3B31_group*	0.73 ^b^	4.54 ^ab^	7.83 ^a^	5.50 ^a^	7.54 ^a^	1.49	<0.01
*Clostridium_sensu_stricto_1*	10.98	0.98	7.51	2.78	2.72	2.33	0.070
*Streptococcus*	2.30 ^ab^	0.65 ^b^	2.82 ^a^	5.28 ^a^	5.47 ^a^	0.95	<0.01
*Agathobactere*	1.02	3.00	4.35	3.05	3.28	1.05	0.16
*Blautia*	3.72	2.65	1.75	3.80	2.00	0.85	0.24
*Terrisporobacter*	7.61	0.46	2.02	0.88	1.48	1.10	0.079

^a,b^ Different superscripts within a row indicate a significant difference (*p* < 0.05). Values are least square means of six replicate pens with four barrows and four gilts per pen per treatment. (i) C, control diet; (ii) EOM1, C + 400 mg/kg EOM1 supplement; (iii) Z, C + 1600 mg/kg ZnO; (iv) ZOM1, Z + 400 mg/kg EOM1 supplement; and (v) ZOM2, Z + 400 mg/kg EOM2; SEM, standard error of the mean.

## Data Availability

The data presented in this study are available on request from the corresponding author. The data are not publicly available due to the requirements of the current project.
